# Global research hotspots and trends of acute rejection after liver transplantation from 1988 to 2022: a bibliometric analysis

**DOI:** 10.3389/fphar.2024.1357468

**Published:** 2024-04-17

**Authors:** Zhiwei Xiong, Zhen Yang, Qiuguo Wang, Ting Li

**Affiliations:** ^1^ Department of Liver Transplantation, The Second Xiang-ya Hospital, Central South University, Changsha, China; ^2^ The Intractable Diseases Diagnosis and Treatment Center for Liver, Gallbladder, Pancreas and Intestine, Department of Hepatobiliary Surgery, Hunan Provincial People’s Hospital, The First Affiliated Hospital of Hunan Normal University, Changsha, China; ^3^ Department of Cardiovascular Surgery, The Second Xiang-ya Hospital, Central South University, Changsha, China

**Keywords:** bibliometric analysis, acute rejection, liver transplantation, Citespace, VOSviewer, immunosuppression

## Abstract

**Background:** Acute rejection (AR) is the predominant form of rejection observed in liver transplantation and plays a crucial role in transplant immunology. This study aims to utilize bibliometric analysis to understand the *status quo*, hotspots, and future trends of research on AR after liver transplantation.

**Methods:** We searched the Web of Science Core Collection (WoSCC) for studies on AR after liver transplantation published from 1988 to 2022. The Bibliometric Online Analysis Platform, VOSviewer, and CiteSpace were used for analysis of all extracted publications.

**Results:** This study included 2,398 articles published in 456 journals by 12,568 authors from 1,965 institutions in 55 countries/regions. The United States and its affiliated institution, the University of Pittsburgh, were the most productive contributors. *Transplantation* (n = 12,435) was the most frequently cited journal. Neuhaus P (n = 38) was the highest output author, and Demetris AJ (n = 670) was the most co-cited author. The research hotspots of AR after liver transplantation include pathogenesis, immunosuppressive therapy, and prognosis. Emerging research directions include regulatory T cells, immunosuppression minimization, intra-patient variability (IPV) of tacrolimus, and novel non-invasive diagnostic markers.

**Conclusion:** Our study utilized bibliometric methods to analyze the study of AR after liver transplantation over the past 35 years. With the prolonged survival of liver transplant recipients, the most active areas currently focus on individualized treatment and improving patient prognosis. Minimizing adverse reactions to immunosuppressive therapy while simultaneously avoiding an increase in the risk of AR remains a future research focus.

## 1 Introduction

In 1963, Starzl TE performed the world’s first case of liver allograft on a child with congenital biliary atresia, but unfortunately, the child died on the operating table due to massive bleeding (Starzl et al., 1964). After that, Starzl TE performed several human liver transplant operations, but the survival time of the recipients did not exceed 1 month. Afterwards, the possible causes of the failure were analyzed, and it was found that AR was one of the most important factors affecting the early prognosis after liver transplantation (Starzl et al., 1968). With the development of organ preservation methods, surgical techniques, and immunosuppressive therapy, especially the application of the new immunosuppressant tacrolimus FK506, the incidence of AR after liver transplantation has been greatly reduced, and the postoperative survival rate and survival time have been significantly improved (Todo et al., 1994; Song et al., 2014). At present, liver allograft has become the only curative treatment for patients with end-stage liver disease. Research on AR has always been a hotspot and frontier in this field, and many scholars have achieved fruitful results. However, due to the systemic and complex nature of AR after liver transplantation, which involves multiple disciplines and fields, a single perspective from clinical analysis or basic research may not provide a comprehensive understanding. Conducting bibliometric analysis might be a promising approach.

The concept of bibliometrics was first introduced by Alan Pritchard in 1969 (Pritchard, 1969). Bibliometric analysis is a method that utilizes mathematical, statistical techniques, and visualization tools to analyze parameters such as publication count, countries/regions, journals, authors, and keywords. By conducting comprehensive analyses of publications in a specific field, bibliometric analysis provides a detailed overview of the knowledge landscape and enables researchers to understand the latest research trends. Compared to other methods such as reviews or meta-analyses, bibliometric analysis offers unique advantages. Common bibliometric software packages include CiteSpace, VOSviewer, UCINET, SciMAT, Pajek, and Bicomb, among which CiteSpace and VOSviewer are the most widely used together (Pan et al., 2018; Vargas et al., 2022).

To the best of our knowledge, no bibliometric study on AR after liver transplantation has been reported globally to date. Researchers have previously analyzed clinical liver transplantation in the past 40 years (Jiang et al., 2022), and immunology-related AR, as an important branch, has not been fully analyzed by the author. Therefore, this article comprehensively analyses the research hotspots and emerging trends of AR after liver transplantation during 1988–2022 by combining bibliometric methods, providing a systematic review, and forming corresponding visual maps. This study provides scholars in this field with the current overall framework of AR after liver transplantation, showing the knowledge base, evolutionary path, research frontiers and future research trends.

## 2 Materials and methods

### 2.1 Data selection

**FIGURE 1:**
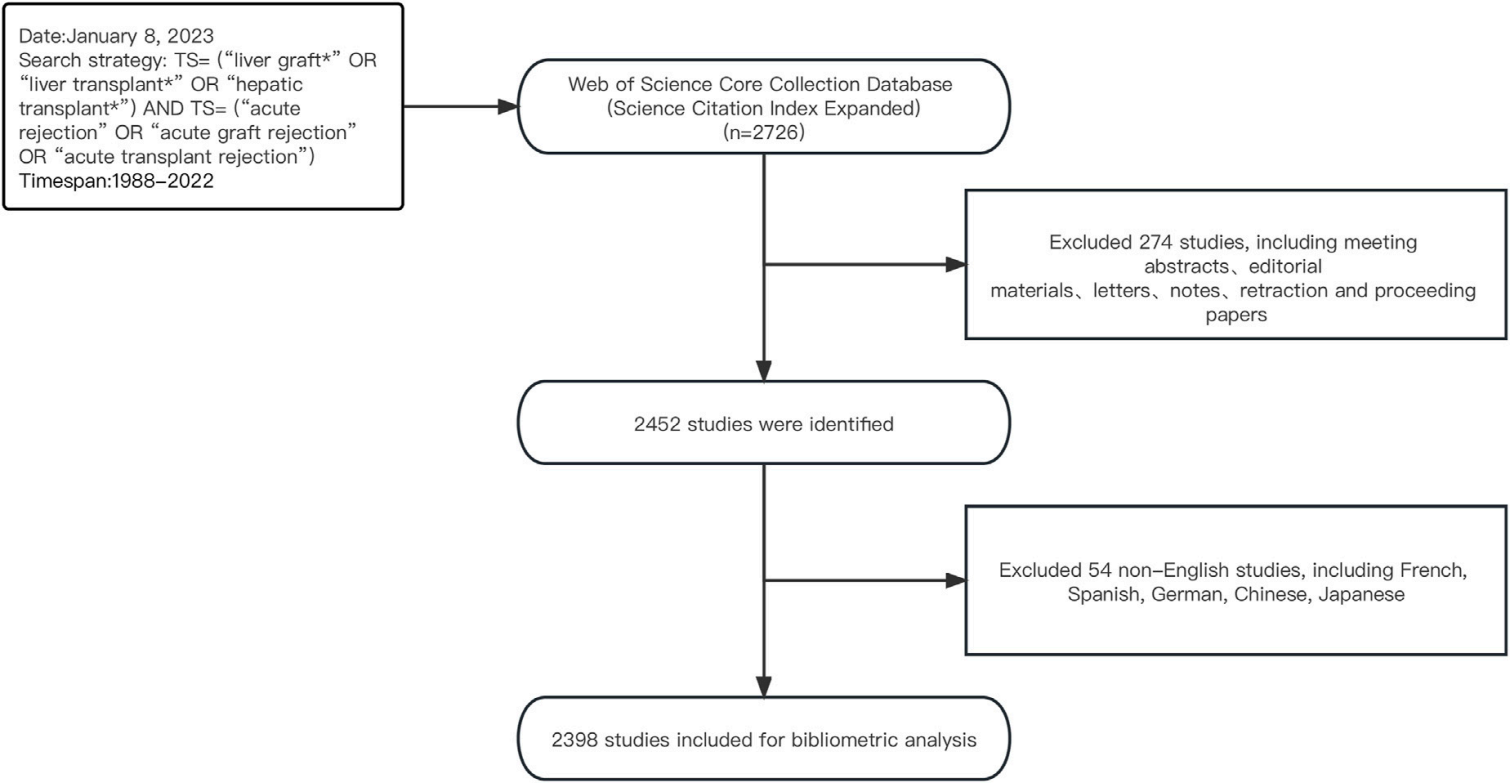
Flowchart of the literature screening process.

As one of the most influential databases for literature, Web of Science (WoS) is widely regarded as the optimal tool for conducting bibliometric analysis, and many researchers have utilized the WoS database to publish bibliometric studies (Thelwall, 2008; Ding and Yang, 2020). Thus, on 8 January 2023, we conducted a search and extracted literature data from the WoSCC database. Using Boolean logical operators, we employed the following search strategy: TS= (“liver graft*” OR “liver transplant*” OR “hepatic transplant*”) AND TS= (“acute rejection” OR “acute graft rejection” OR “acute transplant rejection”). Only literature from the Science Citation Index Expanded (SCI-E) was selected. The retrieval time frame spanned from 1 January 1988, to 31 December 2022, with article types limited to “Article” or “Review” and language limited to English. Ultimately, we analyzed 2,398 articles that met our criteria. A detailed screening process is presented in Figure 1.

### 2.2 Data analysis and visualization

We imported the downloaded data from WoS into the Bibliometric Online Analysis Platform (https://bibliometric.com/app), which enabled us to visualize the annual publication trends and collaboration relationships among different countries/regions. VOSviewer is a free bibliometric mapping software that produces various visual maps such as network and density visualization of institutions, journals, authors, and keywords (Van Eck and Waltman, 2010). CiteSpace, a scientific literature visualization software developed by Professor Chaomei Chen, generates a cluster view and timeline view to summarize the knowledge base and research frontiers of the relevant field (Chen, 2006). This can provide insight into potential research hotspots and trends. In this study, we utilized CiteSpace [version 6.1.R3 (64-bit)] as a supplement to VOSviewer. The parameters in CiteSpace were set as follows: Link Retaining Factor (LRF = 3), Look Back Years (LBY = 5), e for Top N (e = 1), Time Span (1988–2022), Years Per Slice (Starzl et al., 1964), Selection Criteria (g-index: k = 25), and Minimum Duration (MD = 1). The integration of these bibliometric software tools can provide a comprehensive analysis and display of literature data, unveiling various characteristics and trends in the studied field.

## 3 Results

### 3.1 Annual growth trend of publications

**FIGURE 2:**
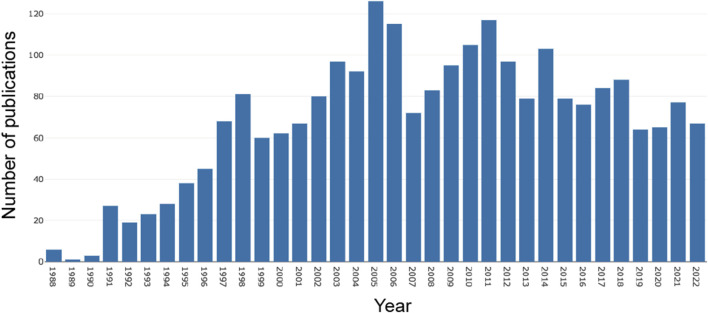
Annual publication trends from 1988 to 2022.

From 1 January 1988, to 31 December 2022, a total of 2,398 publications related to AR after liver transplantation were identified in the WoSCC database. As shown in Figure 2, there were relatively fewer articles on AR after liver transplantation before the 1990s. However, since the 1990s, publications have increased steadily. It reached its peak in 2005 (n = 126) and then experienced a noticeable decline in 2007. However, in the following 5 years, the number of publications steadily increased. From 2011 to the present, there has been a fluctuating downwards trend in the number of publications, but the annual publication count has remained above 60.

### 3.2 Productive countries/regions and institutions

**FIGURE 3:**
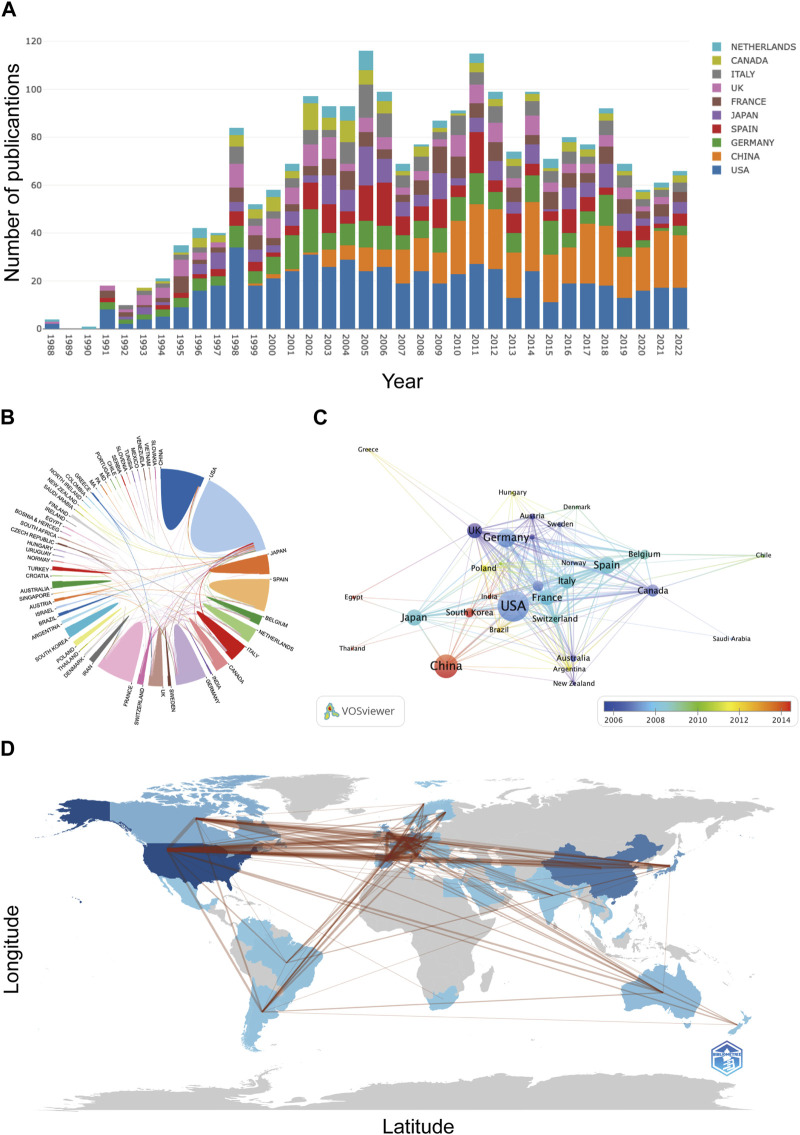
Contributions of countries/regions: **(A)** Annual publication trends of the top 10 countries/regions. **(B)** Collaborative relationship map among countries/regions. **(C)** Overlay map of collaborations among countries/regions. **(D)** Geographical distribution of collaborations among countries/regions.

These 2,398 articles were published by 1,965 institutions from 55 countries/regions. As shown in Table 1, the United States (n = 609), China (n = 363), and Germany (n = 236) are the three countries with the highest cumulative publication volumes. Figure 3A displays the annual publication trends of the top 10 countries in terms of total publication count. Notably, although the United States has long dominated the research on AR after liver transplantation, China (n = 19) surpassed the United States (n = 13) in terms of annual publications for the first time in 2013 and has consistently remained ahead in recent years. Figure 3B illustrates the collaboration between different countries. Publications from the United States and European countries have relatively earlier publication dates, whereas articles from Asian countries, including China, South Korea, and India, have predominantly been published in the last decade (Figure 3C). Figure 3D provides a geographical visualization of the countries involved in research related to AR after liver transplantation.

**TABLE 1:** The top 10 countries/regions and institutions associated with AR after liver transplantation.

Rank	Country/Region	Count	Average citations per publication	Rank	Institution	Count	Average citations per publication
1	United States	609	47	1	Univ Pittsburgh	66	63
2	China	363	12	2	Zhejiang Univ	54	17
3	Germany	236	37	3	Univ Calif San Francisco	45	99
4	Spain	197	32	4	Stanford Univ	34	42
5	Japan	182	22	5	Univ Calif Los Angeles	34	71
6	France	162	39	6	Hannover Med Sch	33	49
7	United Kingdom	161	47	7	Univ Minnesota	32	60
8	Italy	150	33	8	Chongqing Med Univ	31	13
9	Canada	108	40	9	Shanghai Jiao Tong Univ	31	10
10	Netherlands	87	40	10	Univ Toronto	31	33

**FIGURE 4:**
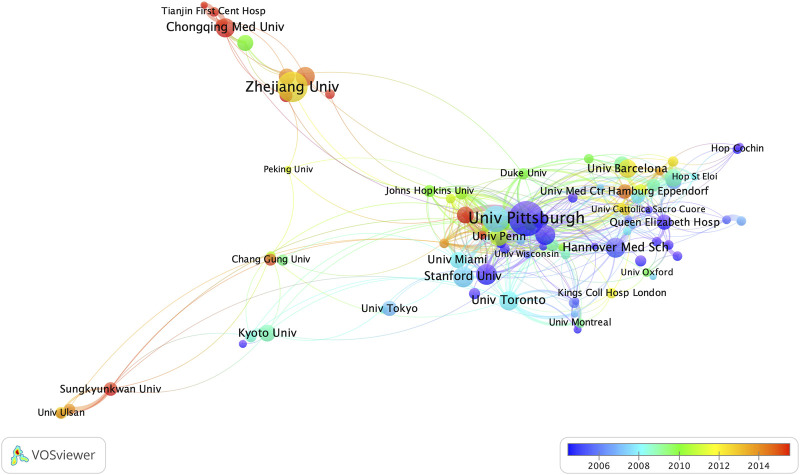
Contributions of institutions. The size of the node represents the number of articles published by each institution, and the redder the color of the node is, the more productive the institution has been in recent years.

For the institutions, the University of Pittsburgh (n = 66) is the most productive institution, followed by Zhejiang University (n = 54) and the University of California, San Francisco (n = 45) (Table 1). The University of California, San Francisco is the institution with the highest average citations per article among the top ten institutions. Among the top ten institutions, five are from the United States, while three are from China. Figure 4 shows the cooperative relationship between institutions. In contrast to collaboration between countries, there is closer collaboration among domestic institutions.

### 3.3 Productive journals and co-cited journals

**FIGURE 5:**
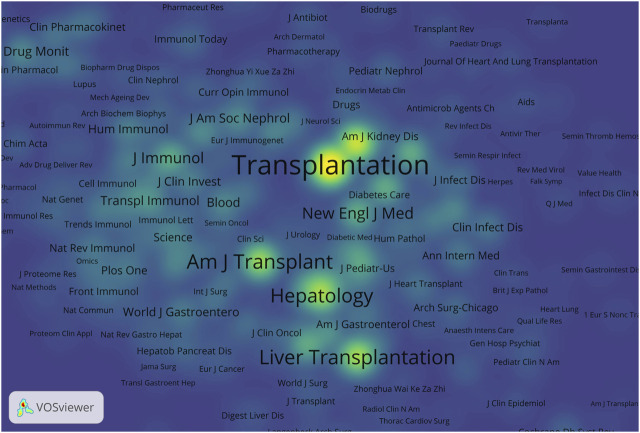
Density visualization of co-cited journals. The size of the word and the opacity of yellow are positively associated with the co-cited frequency.

A total of 456 journals published literature on AR after liver transplantation and cited references from 4,796 journals. Table 2 displays the top 10 journals in terms of publication count and co-cited journals. *Transplantation Proceedings* ranked highest with 334 articles published. Among the co-cited journals, *Transplantation* ranked first by a significant margin and was the only journal with over 10,000 citations. Among all the co-cited journals, a density map was generated for the top 1,000 journals with the highest total link strength, providing a clear visualization of highly cited journals in Figure 5.

**TABLE 2:** The top 10 journals and co-cited journals associated with AR after liver transplantation.

Rank	Journal	Count	Q	Rank	Co-cited journal	TC	Q
1	Transplantation Proceedings	334	Q4	1	Transplantation	12,435	Q1
2	Transplantation	286	Q1	2	Transplant Proceedings	6,432	Q4
3	Liver Transplantation	157	Q1	3	Liver Transplantation	4,521	Q1
4	American Journal of Transplantation	94	Q1	4	Am J Transplant	4,107	Q1
5	Clinical Transplantation	89	Q2	5	Hepatology	3,851	Q1
6	Pediatric Transplantation	78	Q3	6	Lancet	1,577	Q1
7	Transplant International	65	Q1	7	New Engl J Med	1,568	Q1
8	Transplant Immunology	51	Q4	8	Clin Transplant	1,496	Q2
9	Hepatology	43	Q1	9	J Hepatol	1,431	Q1
10	Annals of Transplantation	42	Q4	10	J Immunol	1,368	Q2

TC, total citation; Q Quartile in category.

**FIGURE 6:**
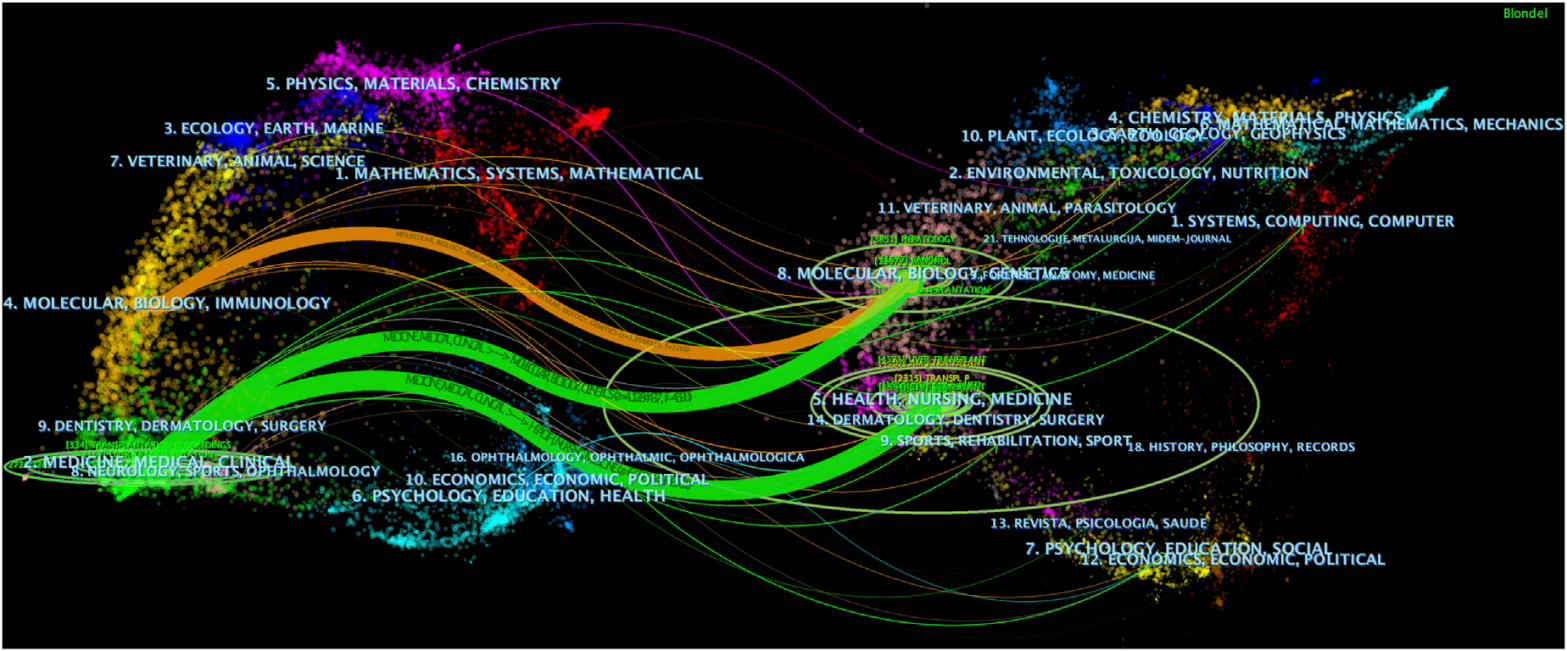
The dual - map overlay of journals (Left: the citing journals; Right: the cited journals).

The dual map overlay feature of CiteSpace was used to display the distribution of journal topics at the disciplinary level, revealing the overall scientific contribution (Chen, 2016). As shown in Figure 6, there are three distinct citation pathways. The two green citation pathways indicate that literature published in Medicine/Medical/Clinical journals primarily cites literature from Molecular/Biology/Genetics and Health/Nursing/Medicine journals. The orange pathway indicates that literature published in Molecular/Biology/Immunology journals primarily cites literature from Molecular/Biology/Genetics journals.

### 3.4 Productive authors and co-cited authors

**FIGURE 7:**
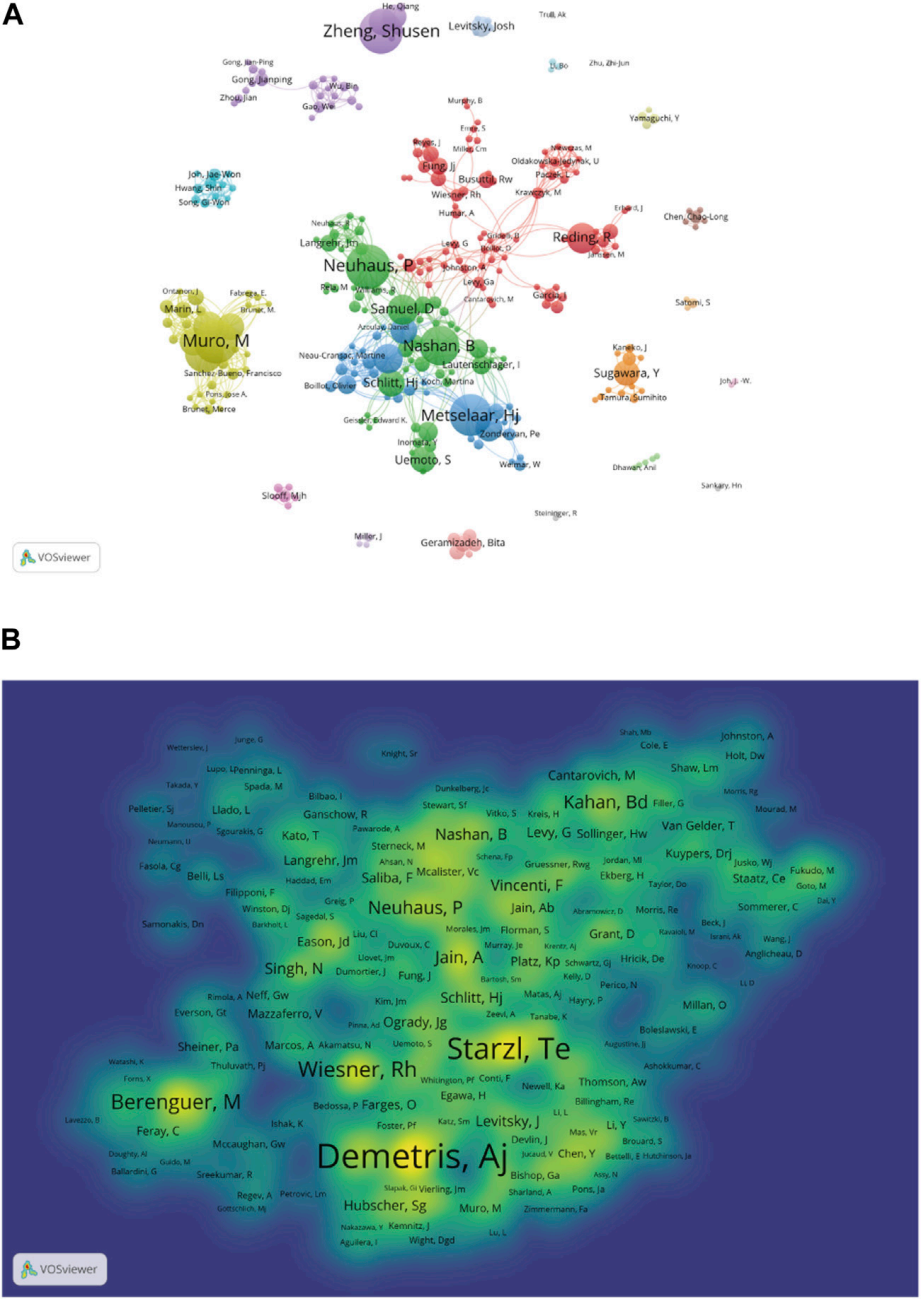
Contributions of authors and co-cited authors: **(A)** Network map of authors. **(B)** Density map of co-cited authors. The size of the node indicates the author’s co-occurrence frequencies while its different colors reflect different clusters, and the links reflect the co-occurrence relationship between authors (Map A).

The bibliometric analysis results showed that 12,568 authors participated in research related to liver transplant rejection, citing a total of 29,629 authors’ articles. As shown in Table 3, Neuhaus P (n = 38) ranked first in terms of publication count, followed by Muro M (n = 37), Zheng SS (n = 37), Metselaar HJ (n = 35), and Nashan B (n = 33). In terms of co-cited authors, the top 10 authors were cited more than one hundred times, with Demetris AJ (n = 670) being the most cited author, followed by Starzl TE (n = 530), Wiesner RH (n = 308), Berenguer M (n = 274), and Neuhaus P (n = 219). Additionally, we used VOSviewer to draw a network map based on the collaboration between authors (Figure 7A). Different colors represent different clusters, there is a closer collaboration between authors within the same cluster. Neuhaus P, Samuel D, and Metselaar HJ from different clusters also have good collaborative relationships. Furthermore, there were 1,366 authors with co-cited exceeding 10, a density map was created for the top 1,000 authors with the highest total link strength (Figure 7B), where the levels of yellow and font size clearly indicate highly co-cited authors. It is evident that Demetris AJ is the most frequently cited author in this field.

**TABLE 3:** The top 10 authors and co-cited authors related to AR after liver transplantation.

Rank	Author	Count	Rank	Co-cited author	TC
1	Neuhaus P	38	1	Demetris Aj	670
2	Muro M	37	2	Starzl Te	530
3	Zheng Shusen	37	3	Wiesner Rh	308
4	Metselaar Hj	35	4	Berenguer M	274
5	Nashan B	33	5	Neuhaus P	219
6	Minguela A	32	6	Kamada N	202
7	Alvarez-Lopez Mr	25	7	Kahan Bd	201
8	Reding R	25	8	Jain A	187
9	Samuel D	25	9	Busuttil Rw	173
10	Saliba F	24	10	Singh N	150

### 3.5 Keyword co-occurrence, clusters, and evolution

**FIGURE 8:**
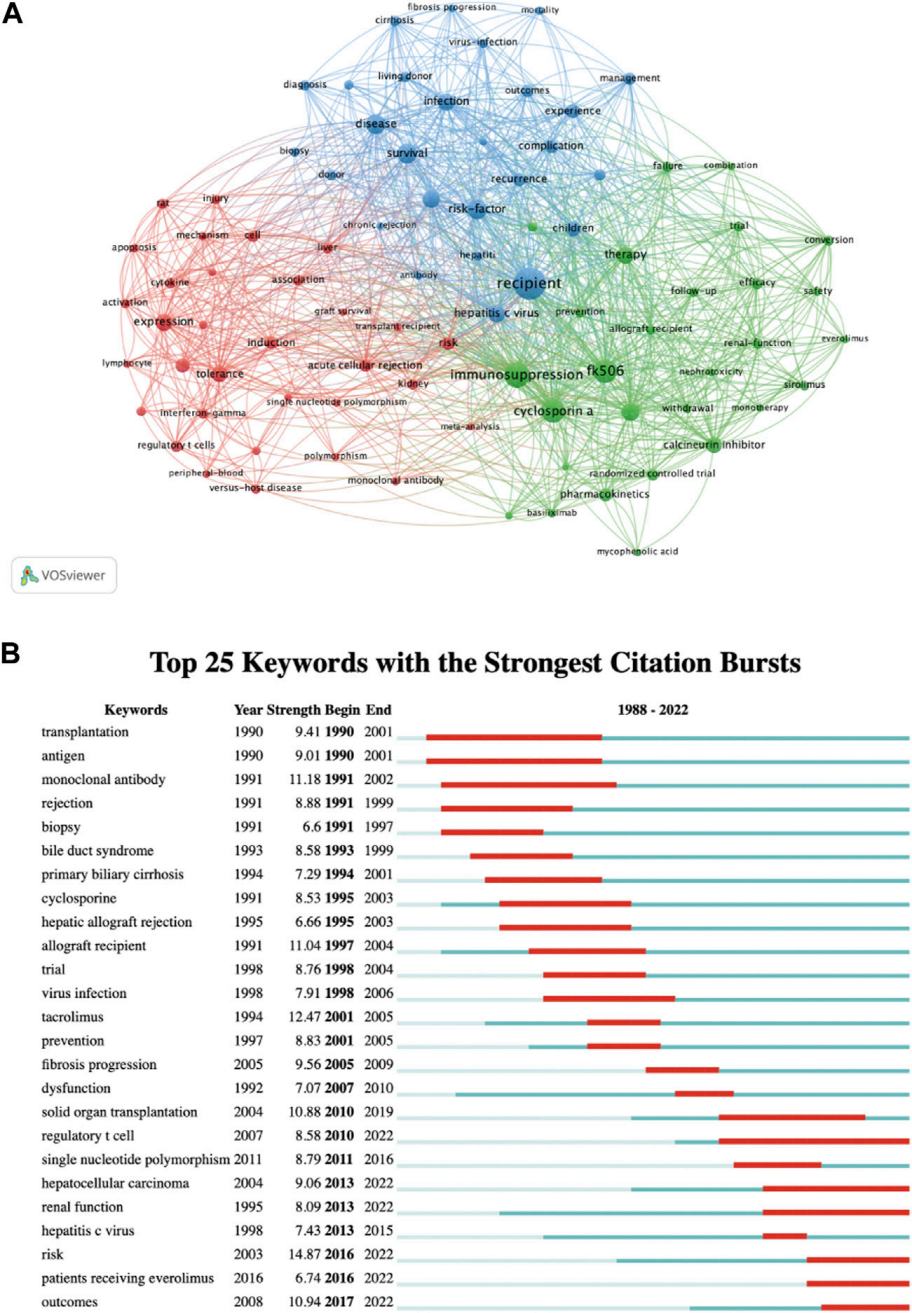
Visualization of keywords: **(A)** Co-occurrence network and clusters of keywords. **(B)** Top 25 keywords with the strongest citation bursts. A red bar indicates high citations in that year and the keywords are ranked by the beginning year of burst (Map B).

A total of 5,779 keywords were extracted by using VOSviewer, and we set a threshold of co-occurrence frequency greater than 5 to generate the keyword co-occurrence network after excluding keywords such as “liver transplantation” and “acute rejection”. Table 4 presents the top 20 high-frequency keywords. As shown in Figure 8A, the keywords can be roughly divided into three clusters: Cluster 1 (32 items, red), Cluster 2 (28 items, green), and Cluster 3 (27 items, blue). In CiteSpace, burst detection reveals keywords that have experienced sudden increases in frequency over time, indicating their significance as active research topics (Chen, 2016). We extracted the top 25 keywords with the strongest citation bursts. As exhibited in Figure 8B, keywords such as “antigen,” “monoclonal antibody,” “biopsy,” and “cyclosporine” emerged as bursts in the early stage of research, while the current research bursts are mainly focused on keywords such as “regulatory T cell,” “hepatocellular carcinoma,” “renal function,” “patients receiving everolimus,” and “outcomes."

**TABLE 4:** The top 20 keywords associated with AR after liver transplantation.

Rank	Keyword	Count	Total link strength	Rank	Keyword	Count	Total link strength
1	Recipient	525	2022	11	Expression	153	518
2	Immunosuppression	333	1,479	12	Hepatitis c virus	151	605
3	Fk506	298	1,300	13	Impact	146	591
4	Cyclosporin a	279	1,110	14	Calcineurin inhibitor	133	647
5	Risk-factor	207	779	15	Tolerance	132	532
6	Survival	201	804	16	Children	122	421
7	Disease	197	671	17	Experience	105	372
8	Mycophenolate mofetil	164	717	18	T-cell	103	342
9	Therapy	156	616	19	Risk	99	367
10	Infection	154	607	20	Pharmacokinetics	98	377

### 3.6 Co-cited references and reference burst

Co-cited reference analysis is a method used to analyze the shared citation relationships among publications, revealing core literature, knowledge structure, and development directions in a research field. The top ten reference articles, ranked by citation frequency, are presented in Table 5. The article by an international panel in 1997 (Banff schema for grading liver, 1997), titled “Banff schema for grading liver allograft rejection: An international consensus document,” has the highest citation count.

**TABLE 5:** The top 10 co-cited references associated with AR after liver transplantation.

Rank	Title	TC	Year	Journal
1	Banff schema for grading liver allograft rejection: an international consensus document	311	1997	Hepatology
2	Acute hepatic allograft rejection: incidence, risk factors, and impact on outcome	116	1998	Hepatology
3	Chronic renal failure after transplantation of a nonrenal organ	107	2003	New Engl J Med
4	Group USMFLS. A comparison of tacrolimus (FK 506) and cyclosporine for immunosuppression in liver transplantation	102	1994	New Engl J Med
5	Randomized trial comparing tacrolimus (FK506) and cyclosporin in prevention of liver allograft rejection	91	1994	Lancet
6	Update of the International Banff Schema for Liver Allograft Rejection: working recommendations for the histopathologic staging and reporting of chronic rejection. An International Panel	63	2000	Hepatology
7	Orthotopic liver transplantation in the rat. Technique using cuff for portal vein anastomosis and biliary drainage	58	1979	Transplantation
8	End-stage renal disease (ESRD) after orthotopic liver transplantation (OLTX) using calcineurin-based immunotherapy: risk of development and treatment	52	2001	Transplantation
9	Liver allograft rejection. An analysis of the use of biopsy in determining outcome of rejection	52	1987	Am J Surg Pathol
10	A surgical experience with five hundred thirty liver transplants in the rat	52	1983	Surgery

**FIGURE 9:**
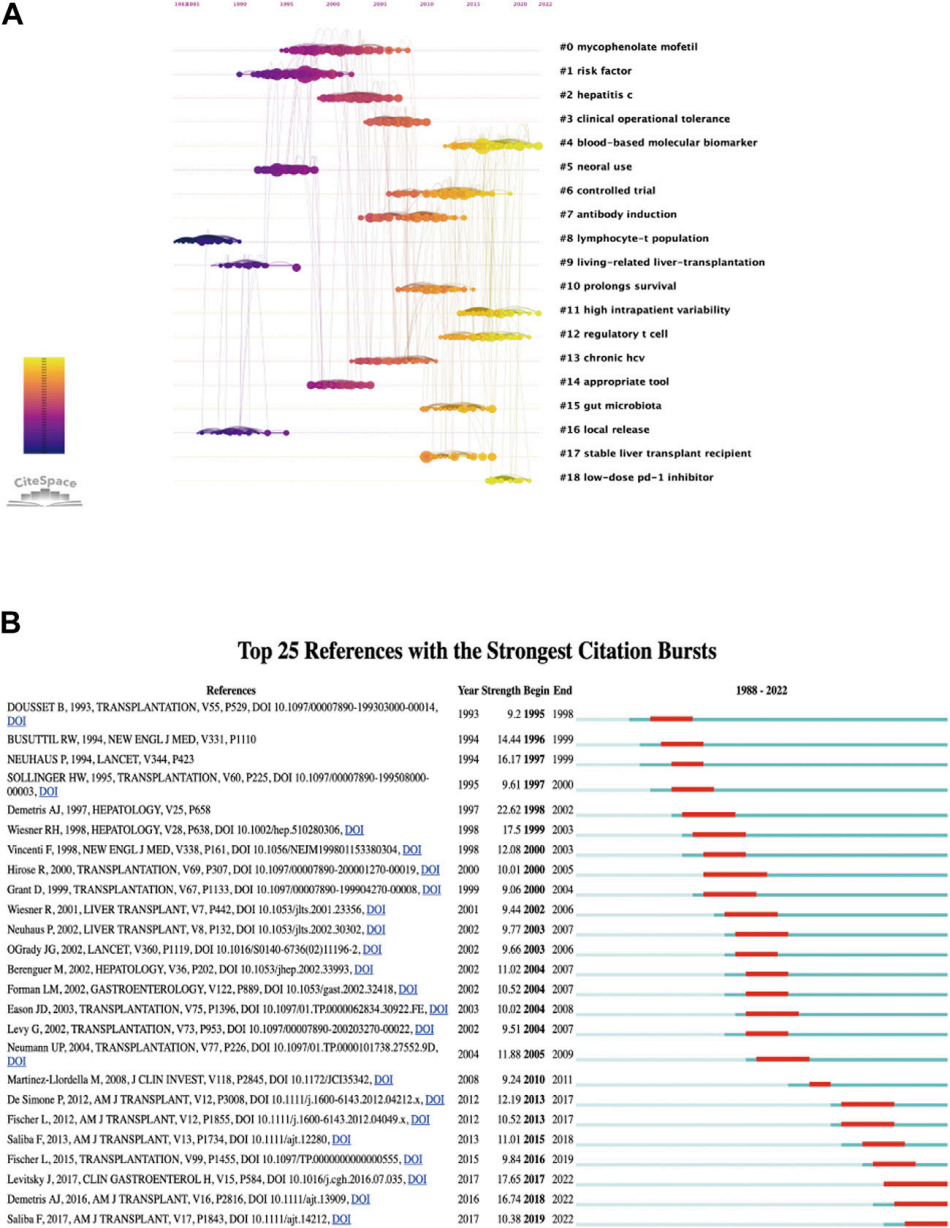
Visualization of references: **(A)** Timeline view and clusters of references. **(B)** Top 25 references with strong citation bursts.

Analysis of the references within the 5 years prior to the publication of the 2,398 articles was conducted to generate clusters that represent research frontiers in different periods. Figure 9A displays a co-cited timeline view clustered using the log-likelihood ratio algorithm. Each node represents a co-cited reference, and the links between nodes indicate that the corresponding references are co-cited within the retrieved set of 2,398 articles. A total of 19 clusters were generated, with the numerical values of the cluster labels negatively correlated with the number of co-cited references in each cluster.

Like keywords, co-cited references can also be analyzed for citation bursts. In this bibliometric analysis, 210 references were found to have citation bursts, and Figure 9B shows the top 25 burst references. It is evident that citation bursts typically occur within 2–3 years after the publication of the referenced papers. From 1995 to 2005, there were multiple papers that experienced citation bursts, and as of 2022, three articles are still in a state of citation bursts.

## 4 Discussion

### 4.1 General information

Using the authoritative literature database, WoSCC, we ultimately extracted 2,398 English papers published in the past 35 years (1988–2022). These papers were authored by 12,568 researchers from 1,965 institutions in 55 countries/regions, published in 456 journals, and cited 48,143 articles published on 4,796 journals by 29,629 authors.

Although the first human liver transplantation was performed as early as 1963, due to the lack of effective immunosuppressive drugs to control AR after surgery, liver transplantation has been controversial (Geissler and Schlitt, 2009). It was not until 1979 that the British scholar Calne RY first applied the immunosuppressive drug cyclosporine to liver transplantation (Williams et al., 1985), that scholars gradually recognized the effectiveness of liver transplantation and promoted the research on AR after liver transplantation. Therefore, as shown in Figure 1, the 1980s was the starting stage of research on AR after liver transplantation. However, with the gradual clinical application of a new type of immunosuppressive drug (such as FK506 and OKT3) in the late 1980s (Millis et al., 1989; Fung et al., 1990), the incidence of postoperative AR greatly reduced, and postoperative survival rate and survival time significantly increased. From the 1990s onwards, research on AR after liver transplantation entered a stage of vigorous development and reached its peak in 2005. However, in the following 2 years, the number of papers published showed a significant decrease, which may be related to new policies for organ transplantation formulated by various countries during this period (Roscam, 2002; Huang, 2007). Since 2011, the number of papers related to AR after liver transplantation has shown a fluctuating downwards trend, but has remained above 60 papers per year, indicating that this field has reached a mature stage.

Both at the national and institutional levels, the United States and its affiliated institutions are the most productive in the field of AR after liver transplantation, and their high average citation count demonstrates their absolute authority in the field (Table 1). The progress made by Asian countries in this field is praiseworthy. However, taking China and its affiliated institutions as an example, although the annual publication volume has increased significantly, the average citation count of articles is relatively low. Although this can be partially explained by the relatively recent publication date of the articles, it is more likely due to concerns about the quality of the papers. This phenomenon is not only limited to this field but is also quite common in other fields. Therefore, for Asian countries such as China to enhance their influence in this field, it is necessary to align with international standards, strengthen cooperation between countries and institutions, and publish more high-quality papers in high-impact journals.

Through subject classification searches in WoS, we found that the articles published in the top ten prolific journals primarily focus on the fields of transplantation and immunology. Analyzing prolific journals allows us to understand the core journals in this field and provides suitable journal choices for researchers to publish their papers. Among the top ten highly cited journals, seven belong to the Q1 zone (Table 2), indicating that high-quality articles published in these journals have significantly contributed to the advancement of the field.

Of the top ten productive authors, nine are from Europe. This finding suggests that European researchers play an important role in the study of AR after liver transplantation. Neuhaus P, as the author with the highest publication volume, mainly conducts clinical research on post-transplant immunosuppressive drugs, particularly calcineurin inhibitors (CNIs), mycophenolate mofetil (MMF), and monoclonal antibodies (Neuhaus et al., 1995; Klupp et al., 1997; Neuhaus et al., 2002). Zheng SS has led multiple studies mainly targeting the prediction of AR after liver transplantation and its influence on prognosis (Wu et al., 2011; Ren et al., 2014; Wei et al., 2018). Muro M’s research mainly focuses on the pathogenesis and risk factors for AR after liver transplantation (Garcia-Alonso et al., 1997; Ontanon et al., 1998; Minguela et al., 2000). Considering co-cited authors, Starzl TE has promoted the clinical application of liver transplantation (Starzl et al., 1964; Starzl et al., 1968). Demetris AJ, Wiesner RH et al. jointly developed histological diagnostic criteria for AR after liver transplantation (Banff schema for grading liver, 1997). The research achievements of these scholars have laid a foundation for the development of AR-related studies after liver transplantation and made significant contributions to the field.

### 4.2 Hotspots and frontiers

Through clustering analysis and burst detection of keywords and references, we can quickly grasp the hotspots and evolutionary paths in the field of AR after liver transplantation over the past 35 years and explore emerging research directions. The research focus on AR after liver transplantation has shifted from early understanding of its pathogenesis to the development and application of immunosuppressive agents. Currently, the mainly focus is on the diversity of individual immune conditions, the development of novel immunosuppressive agents, and the regulation of immune tolerance. In addition, developing personalized immunotherapy plans to reduce complications caused by immunosuppression and improve long-term outcomes for patients may be one of the important directions for future research in the field of organ transplantation.

#### 4.2.1 Pathogenesis and diagnosis of AR after liver transplantation

In the 1970s, with advancements in surgical techniques and organ preservation, the success rate of liver transplantation significantly improved. However, due to the high incidence of AR after surgery, the overall one-year survival rate remained below 30% (Zarrinpar and Busuttil, 2013). After in-depth research, scientists discovered that the recognition and attack of allogeneic antigens by the immune system are the core of AR (Donaldson et al., 1993). CD4^+^ T cells and CD8^+^ T cells play a crucial role in AR, participating in immune responses through the production of cytokines and direct cytotoxic pathways (McCaughan et al., 1990; Adams et al., 1993; Hoffmann et al., 1993). Regulatory T cells have become a recent research focus as they inhibit the activation and function of other immune cells, thereby suppressing immune responses and playing a significant protective role in immune balance regulation (Han et al., 2020; Tanimine et al., 2020). Furthermore, the role of neutrophils in post-liver transplant complications is increasingly gaining attention (Liu et al., 2022a). Research indicates that neutrophil extracellular traps (NETs) released by neutrophils can induce inflammatory reactions, exacerbating the severity of AR after liver transplantation by promoting Kupffer cell M1 polarization and HMGB1 intracellular translocation (Liu et al., 2022b). Therefore, NETs are considered a potential novel therapeutic target for AR after liver transplantation.

Under the assault of the immune system, liver biopsy exhibits characteristic changes such as damage to bile ducts, endothelial cells, and hepatocytes, as well as the infiltration of inflammatory cells (Snover et al., 1987; Schlitt et al., 1991). However, liver biopsy is invasive, expensive, and can lead to complications. Researchers are actively seeking non-invasive biomarkers. Through techniques such as single-cell sequencing and proteomic profiling, researchers have discovered biomarkers such as DSA, donor-derived cell-free DNA, CXCL10, and microRNA that can be used to predict the occurrence and severity of AR (Bonaccorsi-Riani et al., 2016; Shaked et al., 2017; Jucaud et al., 2019; Levitsky et al., 2020). These new findings deepen our understanding of AR after liver transplantation and provide an earlier and safer molecular diagnostic approach (El et al., 2023).

#### 4.2.2 Immunosuppressive therapy in liver transplantation

Due to limited understanding of AR, early prevention of AR relied on empirical use of adrenal corticosteroids (such as prednisone) and nitrogen mustard drugs. However, the effectiveness of these medications in preventing rejection was suboptimal, and the occurrence of adverse reactions was a significant concern. With a deeper understanding of transplantation immunology, researchers began developing more specific and effective immunosuppressive drugs. In the 1980s, immunosuppressive agents such as Cyclosporine A and FK506 were introduced, which significantly improve the prognosis of liver transplant recipients by inhibiting the activity of T lymphocytes and reducing the occurrence of rejection. Subsequently, researchers continuously explored more personalized and targeted immunosuppression strategies. Novel immunosuppressive drugs, including anti-proliferative/metabolic agents (such as MMF) and mTOR inhibitors (such as sirolimus and everolimus), have been introduced into the immunosuppressive regimens (Klupp et al., 2005; Saliba et al., 2017). This has greatly enhanced the long-term survival rate of liver transplant recipients.

#### 4.2.3 Management of long-term complications after liver transplantation

With the extended survival of liver transplant recipients, there is a growing emphasis on improving quality of life. Researchers and clinicians are increasingly concerned about the management of long-term complications, particularly the adverse effects associated with the prolonged use of immunosuppressive agents. These effects mainly include opportunistic infections, renal dysfunction, neurological damage, and recurrence of malignancies, which are major factors affecting long-term survival of patients (Nicastro et al., 2017; Saliba et al., 2019; Qiao et al., 2021). To address these issues, researchers have focused on immunosuppression minimization strategies (Levitsky and Feng, 2018). They have found individual variations in the response to immunosuppressive agents among recipients, and even observed transplant tolerance in some cases (Adams et al., 2015; Kim et al., 2016; Kuypers, 2020). Researchers are also continuously developing new immunosuppressive drugs to replace corticosteroids and CNIs, aiming to reduce their nephrotoxicity and neurotoxicity. For liver transplant patients with malignancies, clinicians have shifted their approach, using immune checkpoint inhibitors (such as Programmed Cell Death 1 Inhibitor) to prevent tumor recurrence (Jin et al., 2022). However, it is important to be cautious about the potential risk of AR when implementing these strategies.

## 5 Strengths and limitations

Compared to traditional reviews, bibliometric analysis provides a more comprehensive overview of the literature in a research field by covering many literature resources. The use of objective metrics and analysis methods enhances the reliability of the conclusions. However, our study also has some limitations. First, we only extracted literature from the SCI-E of the WoSCC database, while other important databases such as PubMed and Scopus were not included. Additionally, we imposed restrictions on the time, language, and types of literature, which may have resulted in the omission of important studies. Second, bibliometric analysis usually focuses on the analysis of quantity and trends, but it has limitations in providing in-depth understanding and interpretation of the research content. It cannot replace systematic reviews and analyses of specific research questions. Nevertheless, bibliometric visualization analysis undoubtedly provides scholars with a more convenient way to understand the research topics, hotspots, and their evolution of AR after liver transplantation. It also provides guidance for exploring valuable research directions.

## 6 Conclusion and future perspective

Acute rejection, as the most common type of transplant rejection, is a crucial aspect of research in transplantation immunology. In this comprehensive bibliometric analysis, we examined the literature related to AR after liver transplantation published from 1988 to 2022. Our analysis revealed the main countries, institutions, journals, and authors in this research field, as well as the knowledge base and evolving hotspots. The results showed that this field has matured after 35 years of development. The latest research topics and hotspots are centered around the management of adverse reactions to immunosuppressive agents, immunosuppression minimization, IPV of tacrolimus, and novel non-invasive diagnostic biomarkers. We anticipate that in the future, with the prolonged survival of transplant recipients, individualized treatment plans and improving patient prognosis will remain the key focus of research. Attaining a subtle balance between optimal immunosuppression and minimal side effects is an urgent issue.

## Data Availability

The original contributions presented in the study are included in the article/supplementary material, further inquiries can be directed to the corresponding authors.

## Abbreviations

AR, acute rejection; CNIs, calcineurin inhibitors; DSA, donor-specific antibody; IPV, intra-patient variability; MMF, mycophenolate mofetil; WoS, Web of Science; WoSCC, Web of Science Core Collection.
